# Expression of P16INK4a in Uveal Melanoma: New Perspectives

**DOI:** 10.3389/fonc.2020.562074

**Published:** 2020-10-13

**Authors:** Daniela Russo, Rosa Maria Di Crescenzo, Giuseppe Broggi, Francesco Merolla, Francesco Martino, Silvia Varricchio, Gennaro Ilardi, Alessandra Borzillo, Raffaella Carandente, Sara Pignatiello, Massimo Mascolo, Rosario Caltabiano, Stefania Staibano

**Affiliations:** ^1^Department of Advanced Biomedical Sciences, Pathology Section, University of Naples Federico II, Naples, Italy; ^2^Department G.F. Ingrassia, Section of Anatomic Pathology, University of Catania, Catania, Italy; ^3^Department of Medicine and Health Sciences “V. Tiberio”, University of Molise, Campobasso, Italy; ^4^Department of Neuroscience, Reproductive Sciences and Dentistry, University of Naples Federico II, Naples, Italy

**Keywords:** uveal melanoma, P16INK4a, CDKN2A, cutaneous melanoma, retinoblastoma

## Abstract

Uveal melanoma (UM) is the most common intraocular tumor in adults. Despite sharing the name and similar morphological features with cutaneous melanoma (CM), it is an entirely different neoplasia with a particular genetic background and clinical behavior. CDKN2A is a gene located at chromosome 9p21, encoding for P16INK4a and P14(ARF) proteins, whose role as a tumor suppressor has been clearly defined in many malignant tumors. CDKN2A frequently presents germline mutations in familial CM and epigenetic downregulation in a considerable percentage of sporadic CM. It has been hypothesized that CDKN2A alterations are early events in CM development, playing a central role in the malignant transformation of melanocytes. Alterations of the CDKN2A gene reduce the expression of P16INK4a in most CM subtypes. Immunohistochemical evaluation of P16INK4a is currently used, in association with Ki67 and HMB45, in pathology practice to discriminate between dysplastic nevi and melanoma. On the other hand, CKDN2A is rarely mutated in UM, and the immunohistochemical expression of P16INK4a has only been reported in small case series. We tested P16INK4a expression on paraffin-embedded tissue sections from 9 tissue microarrays (TMAs), built with 2 mm cores derived from 133 uveal melanoma FFPE blocks, collected from 1990 to 2018, and from selected paraffin-blocks of 3 UM liver metastases. The immunohistochemical expression of P16INK4a was assessed with a visual evaluation by light microscopy and then with a digital approach. Both approaches, with an acceptable concordance rate, revealed P16INK4a expression in a large proportion of UM cases and all liver metastases, opening new possibilities of using it in the differential diagnosis between cutaneous and uveal melanoma metastases in cases of unknown primary tumor or patients with two different primary melanomas.

## Introduction

Uveal melanoma (UM) is rare ocular neoplasia with a steady incidence rate in Europe (5–7 cases per million) (1). UM originates from melanocytes of the uvea, the pigmented central concentric layer of the eye. The eye is the second most common site of occurrence of melanoma following the skin, and UM represents 5% of all melanomas (2). Despite its morphological similarity with cutaneous melanoma (CM), UM is characterized by a distinct genetic profile and unique biological and clinical behaviors (3). Knowledge related to UM has grown in recent years, with improved characterization of its molecular background and the relationship between specific genetic alterations and prognosis (4). Up to 50% of UM patients develop metastases, mostly in the liver (5). In the last few years, research has focused on prognostic evaluation systems aimed at tailoring patient follow-up according to metastatic risk. UM has few recurrent mutations, but initiating mutations of either GNAQ or GNA11 are present in more than 80% of UM in a mutually exclusive manner. GNAQ and GNA11 genes encode for the alpha subunit of the G protein, and their mutations cause the activation of MEK, protein kinase C and YAP/TAZ pathways. About 20% of UM present the L129G-activating mutation in the CYSLTR2 gene, or the D630Y mutation in the PLCB4 gene, both acting in the same pathway of GNAQ and GNA11. GNAQ, GNA11, CYSLTR2, and PLCB4 somatic mutations are crucial in early UM development but are not related to metastatic behavior or a bad prognosis (6). Therefore, these mutations are currently considered as precursor events in UM development, with the need for a “second hit” to complete the malignant transformation (7).

Loss of chromosome 3 and mutations of the BAP1 gene are strictly related to UM progression. Monosomy of chromosome 3 is the most frequent chromosomal alteration occurring in 50–60% of UM and is the strongest known predictor for the subsequent development of metastases. Monosomy of chromosome 3 is frequently associated with a gain of 8q, further increasing metastatic risk. Conversely, the presence of 6p amplification represents a “protective” factor, because of its association with a good prognosis and low metastatic risk (2). The BAP1 gene is located on chromosome 3 (3p21.1), encodes a nuclear ubiquitin carboxy terminal hydrolase with deubiquitinase activity and tumor suppressor functions. BAP1 inactivating mutations occur in 47% of primary UM and 84% of metastatic UM cases and are probably related to the loss of cellular differentiation and the acquisition of stem cell features (2). All UM with BAP1 mutations also have monosomy of chromosome 3. UM may present mutations of the SF3B1 gene, mostly in the absence of BAP1 mutations, and in 22% of cases are associated with the loss of chromosome 3. In the absence of chromosome 3 monosomy and BAP1 mutations, missense mutations in the amino-terminal part of the EIF1AX gene may be identified. About 18% of UM present mutations of the EIF1AX gene, associated with low metastatic risk (6).

Uveal melanoma never present BRAF mutations (3), which, however, are reported in 50% of CM (8). BRAF mutated CM have shown a sensitivity to BRAF inhibitors that are used as first-line therapy in metastatic or unresectable BRAF mutated CM (9), also in association with MEK inhibitors (10). Unfortunately, neither this association nor the single-use of MEK inhibitors correlate to a significant improvement of overall survival in patients with metastatic UM (3), once again highlighting the biological differences between these two tumors. BRAF wild-type CM may present other mutations such as N-Ras, K-Ras, or H-Ras mutations (observed in 25% of CM), NF1 mutations (in 15% of CM), as well as alterations of the TERT-promoter or tumor suppressor genes such as ARID2, TP53, PTEN and also CDKN2A (8). The CDKN2A gene is a tumor suppressor gene located on chromosome 9 band 21.3; it encodes for several transcript variants, which differ in their first exons, and two major proteins: P16INK4a, which is a cyclin-dependent kinase inhibitor, and p14ARF, which binds the p53-stabilizing protein MDM2 (11). P16INK4a, through the inhibition of CDK4 and CDK6 (cyclin-dependent kinases 4 and 6), activates the retinoblastoma protein (RB), which blocks cellular cycle progression from phase G1 to phase S (12). CDKN2A mutations are commonly found in familial melanoma (13), and it has been hypothesized that alterations in cell cycle control genes are necessary for the acquisition of invasive potential and the transformation into invasive melanoma (8).

CDKN2A mutations determine the alteration of protein P16INK4a expression. Many authors have demonstrated that P16INK4a immunohistochemical expression is preserved in benign nevi and is lost in CM (14), except for desmoplastic melanoma (12). While P16INK4a has been shown to be of little use when used alone; a panel encompassing P16INK4a, Ki67, and HMB45 is more effective in the differential diagnosis of melanocytic lesions in clinical practice (15). According to recent studies, P16INK4a, Ki67, and HMB45 immunohistochemistry could be considered as a first-line tool in melanocytic tumor diagnosis, followed by cytogenetic tests (16).

CDKN2A mutations have rarely been described in UM (17), and there are few studies (18, 19) concerning the immunohistochemical evaluation of P16INK4a. Merbs et al. showed that p16 inactivation by homozygous deletion or methylation occurs in 27% of UM (20), it was also shown that the p16(INK4a) promoter is hypermethylated in 6 out of 12 UM cell lines and in 7 out of 22 primary UM (21). The activation of INK4A is required for efficient melanocyte differentiation. It has been shown that INK4A can be activated by different factors, including MITF, a protein also required for maintaining INK4A expression in mature melanocytes. MITF binds the INK4A promoter, activates p16(Ink4a) mRNA and protein expression, and induces retinoblastoma protein hypo-phosphorylation, thereby triggering cell cycle arrest (22). Thus inactivation of CDKN2A through methylation of the promoter or homozygous deletion could be part of the development of a proportion of UM (23). Moreover, recent findings identified a particular association between the deletion of CDKN2a and 8q amplification in a parallel stepwise fashion. It was hypothesized that the monoallelic deletion of CDKN2A and the gain of at least three copies of 8q are early events in UM development. Conversely, acquisition of biallelic loss of CDKN2A and higher amplification of 8q could be relevant in metastatic progression (24, 25).

The retinoblastoma (RB) pathway is also crucial in UM evolution. The RB gene has never been found mutated in UM, but the protein is often phosphorylated at residues of the COOH region, and probably this phosphorylation could interrupt the RB tumor suppressor function (19). There is evidence that also the p53 pathway is probably inactivated in UM through MDM2 overexpression (26).

## Materials and Methods

### Case Series and Study Population

Formalin-fixed, paraffin-embedded tissue blocks of 133 UM were collected. All patients included in this study underwent enucleation between 1990 and 2018. We retrieved the specimens from the archives of the Pathology Section of the Department of Advanced Biomedical Sciences, University of Naples “Federico II,” and of the Department G.F. Ingrassia, Section of Anatomic Pathology, University of Catania. TMAs were cored all together at the same time.

We excluded 6/133 cores due to core loss during processing. We ran the visual analysis on 127 cores, for Digital Image Analysis, 7/127 cores were excluded due to poor performance at the quality check, thus 120/127 cores underwent digital p16 expression assessment (The workflow is summarized in the flow-chart below).

The clinical data and pathological features of the tumors are reported in Table 1. Updated follow-ups were available for 123 cases. We also assessed P16INK4a expression on three histological samples from surgical resection of liver metastases from UM.

**TABLE 1:** Clinicopathological features of the study population.

			% of the total
**Age**	Min	19	
	Max	90	
	Mean	63.70	
	Median	65.50	
**Gender**	Female	69	52%
	Male	64	48%
**Site**	Choroid	110	83%
	Ciliary body, choroid	17	13%
	Iris, Ciliary body	1	1%
	Missing	5	4%
**Cellularity**	E	34	26%
	MIX	57	43%
	S	40	30%
	Missing	2	2%
**LBD (mm)**	Min	2.2	
	Max	37	
	Mean	14	
	Median	14	
**Pre-Surgery therapy**	No	108	85%
	Yes	19	15%
	Total	127	100%

The study was performed according to the guidelines of the Institutional Ethic Committee, which, in agreement with Italian law concerning the topics of the current research and according to the Declaration of Helsinki, require, for studies based only on retrospective analyses on routine archival FFPE-tissue, a written informed consent from the living patient, following the indication of Italian DLgs No. 196/03 (Codex on Privacy), as modified by UE 2016/679 law of the European parliament and Commission at the time of surgery.

### Tissue Microarray (TMA)

Hematoxylin-Eosin (H-E) sections of all UM cases were reviewed by expert pathologists (SS, DR, RMdC) and, for each case, the most representative areas were selected, excluding hemorrhagic, necrotic or, if possible, hyperpigmented ones and considering intra-tumor heterogeneity (27). Three-mm cores, derived from the most representative areas of each tumor (from 2 to 3 cores per tumor depending on tumor size), were taken by a manual tissue-array instrument (Tissue-Tek Quick-Ray, Sakura Finetek, Torrance, CA, United States). The tissue cores were put into empty “recipient” paraffin blocks with 30 holes each. Subsequently, the recipient blocks were placed on metal base molds. The paraffin-embedding was performed as follows: the blocks were heated at 42°C for 10 min and their surface was flattened by pressing a clean glass slide on them. We obtained nine TMAs. Two 4-μm sections were cut from each TMA and from 3 selected paraffin blocks of liver metastases with a standard microtome. The first section was stained with H&E to confirm the correct execution of the procedure (presence and integrity of tumor cores).

### Immunohistochemistry

A 4-μm tissue section from each TMA was transferred onto TOMO^®^ IHC Adhesive Glass Slides (Matsunami Glass Ind., Ltd., Japan), for the immunohistochemical evaluation of P16INK4a, following the standard procedure described below and in agreement with the literature evidence about environmental conditions that could reduce the immunoreactivity of samples (28, 29). After heating to 55°C for 60 min, immunostaining for P16INK4a was performed with the fully automated Ventana Benchmark Ultra platform (Ventana Medical Systems Inc., Tucson, AZ, United States) using the CINtec P16INK4a kit (Roche MTM laboratories AG, Heidelberg, Germany). The tissue paraffin sections were deparaffinized and subjected to antigen retrieval using CC1 buffer for 30 min. Subsequently, they were consecutively incubated in the prediluted CINtec p16 primary antibody (clone E6H4) for 20 min at room temperature and revealed with Ultra View Universal Alkaline Phosphatase Red Detection Kit (Ventana Medical Systems, Inc., Tucson, AZ, United States). Slides were counterstained with Hematoxylin II for 8 min (Ventana Medical Systems, Inc., Tucson, AZ, United States) and Bluing reagent for 4 min and then washed. A section of a melanocytic nevus with P16INK4a high expression was used as a positive control. The primary antibody was omitted from negative controls.

The immunohistochemical staining for P16INK4a was evaluated by expert pathologists, positive cases were considered the ones that showed red nuclear and/or cytoplasmic staining, visual categories describing P16INK4a positivity in tested samples were as follows: “negative” = no visually detected positivity in the tumor; “low”: <10% positive tumor cells; “intermediate”: 10–40% positive tumor cells; “DP” (Diffuse Positive): >40% positive tumor cells (18).

### Glass Slide Digitalization and Digital Image Analysis

H&E and Immunostained TMA slides were digitalized with an Aperio AT2 digital pathology slide scanner at 40× (Leica Biosystems Nussloch GmbH, Heidelberger, Germany).

The slides were analyzed using QuPath (30), an Open-Source software that allowed us to perform digital image analysis through tissue and nuclei segmentation, and to compute cellular features with various algorithms automatically. Then we disarrayed our TMAs and used the “Tissue Detection” to define the region of interest (ROI) containing tissue within the core. To perform digital quantization of P16INK4a positivity, we first performed a color deconvolution step. Three bounding boxes containing few pixels of a single color (hematoxylin, alkaline phosphatase, melanin) each were made, and the QuPath-embedded color sampling tool was used to define color channels. Following a quality control step, discarding damaged cores and cores containing artifacts, to select only evaluable cores, cell detection was performed using the QuPath “positive cell detection” tool with standard parameters but adjusting the positivity threshold on the basis of the weakest positive nuclei. A tumor segmentation step was not included because of the nature of our TMAs, which were built so that each core is totally or mostly occupied by tumor areas.

### Statistical Analysis

SPSS software (IBM Corp. Released 2013. IBM SPSS Statistics for Windows, Version 25.0. Armonk, NY, United States) was used for statistical analysis. Survival analysis was performed testing the differences between Kaplan–Meier survival curves with the log-rank test. The statistical significance of P16INK4a distribution between cellularity groups was tested by ANOVA statistical test between groups. The correlation between P16INK4a and LBD was tested by Pearson correlation test.

Statistical tests were considered significant for values of *p* < 0.05.

### Design of the Study

A detailed workflow of the study design has been provided as Supplementary Figure 1.

## Results

### TMA Preparation and Visual Assessment of P16INK4a Tissue Expression Study Population

We assessed P16INK4a expression and cellular localization in a cohort of 127 UM samples selected from the archives of Pathology Unit of University “Federico II” of Naples and from the Department G.F. Ingrassia, Section of Anatomic Pathology, University of Catania, with validated follow-up, arranged in tissue microarrays (TMA). The clinicopathological features of the study population are reported in Table 1.

All the analyzed samples were primary tumors; we visually evaluated 3 more samples from UM liver metastases.

Two blinded pathologists assessed the P16INK4a expression by visually evaluating the immunostaining at the microscope; they categorized the evaluated samples into four distinct categories labeled “Negative,” “Low,” “Intermediate,” and “DP” (Diffusely positive) based on the percentage of positive tumor cells (see section “Materials and Methods”). The discordant cases were discussed until an agreement was reached.

We found P16INK4a positivity at immunostaining in 98 out of 127 cases of primary UM tumors and in 3 out of 3 metastases. In 29 cases, we did not observe P16INK4a expression evaluating UM samples at the microscope.

**FIGURE 1:**
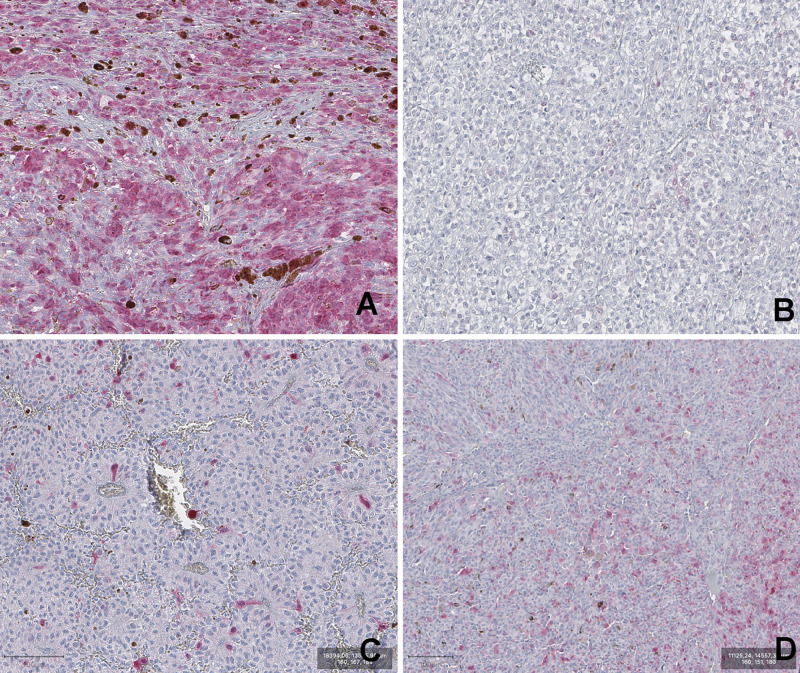
Representative images of P16INK4a IHC staining of Uveal melanoma samples. **(A)** DP (diffuse positivity). **(B)** negative. **(C)** low. **(D)** intermediate. (magnification 100×).

A summary of frequency distribution of visual categories of P16INK4a expression in UM is reported in Table 2. Representative images of the four visual categories are shown in Figure 1.

**TABLE 2:** Frequency distribution of UM cases across the visual categories (DP: diffusely positive).

Visual categories	Frequency N (%)
DP	26 (20%)
Intermediate	32 (25%)
Low	40 (31%)
Negative	29 (23%)
Total	127 (100%)

### Digital Image Analysis of p16INKa Tissue Expression in Uveal Melanoma Samples

We used the QuPath digital platform to quantify the percentage of P16INK4a expression in UM tumor cells.

Following digital image acquisition of P16INK4a immunostained UM glass slides, we processed the virtual slides with the QuPath software.

In the first instance, we estimated the stain vector; afterward, we disarrayed the TMAs isolating each core to treat them as single objects. The “Positive Cell Detection” algorithm was applied to detect single nuclei through Hematoxylin counterstaining, simultaneously indicating the percentage of cells positive to the stain vector of interest. The algorithm allowed us to obtain a quantitative measure of P16INK4a expression in the evaluated cores.

A detailed report of the whole digital quantitation is reported in Table 3. The Digital Image Analysis approach included a more stringent quality check protocol that excluded 7 cores out of the 127 considered for visual evaluation.

**TABLE 3:** Summary of Digital Image Analysis (DIA) based on the evaluation of p16 expression.

N	Valid	120
	Missing	7
**Digital Image Analysis**		
Mean		23.35
Median		18.89
Std. Deviation		22.47
Range		89.80
Minimum		0.00
Maximum		89.80

*(Missing: cases excluded from the digital analysis because cores did not pass the digital quality check).*

Overall, the digital image analysis confirmed the evidence of expression for P16INK4a protein in a large proportion of UM.

### Comparison of Visual Evaluation With Digital Image Analysis Quantification

**FIGURE 2:**
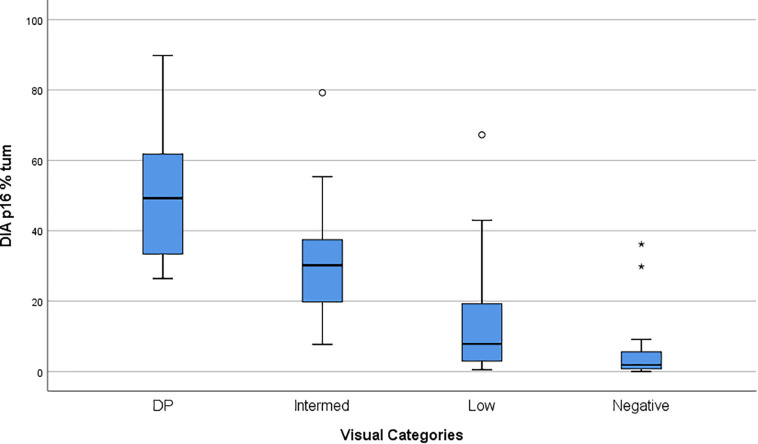
Box Plot showing the P16INK4a Visual Categories compared to P16INK4a expression quantified by DIA (Digital Image Analysis). Values on the *Y*-axis are% of UM tumor cells positive to P16INK4a.

**FIGURE 3:**
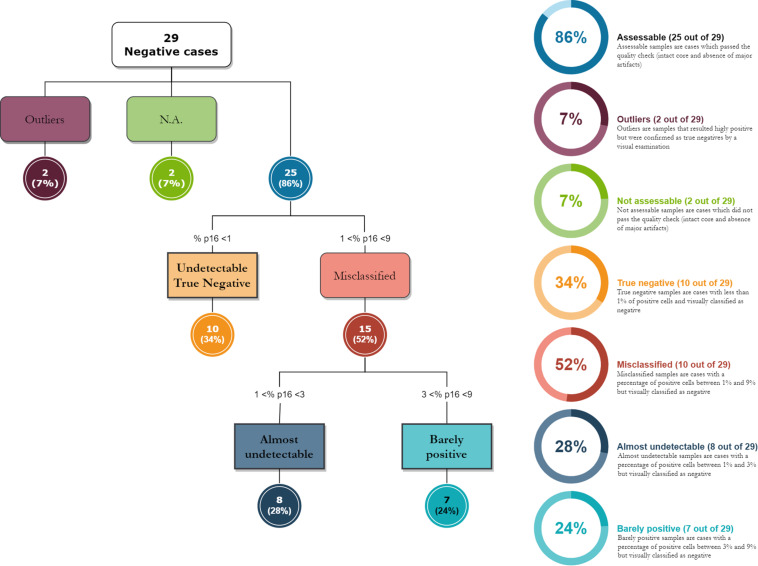
The diagram shows how digital image analysis has highlighted the presence of weakly positive cases in the “negative” group, as assessed under a microscope. The digital evaluation is extremely sensitive and allows the pathologist to evaluate a signal positivity that is barely visible to the naked eye.

We compared the Visual evaluation to the Digital assessment of P16INK4a tissue expression in our study population. Table 4 shows the distribution of the cases by DIA in the groups corresponding to the categories found at the visual evaluation. Figure 2 shows a box plot with the percentage of P16INK4a positive tumor cells grouped by Visual categories. The picture shows a significant distribution of DIA P16INK4a data in the visual categories, although we managed to identify some outliers. A more detailed statistical analysis of digital assessed p16 percentage value distribution across Visual categories is shown in Table 5. The “negative” manually evaluated cases were originally 29, out of which two cases did not pass the quality check for Digital Image Analysis, two other cases turned out to be outliers since they were hyperpigmented cases whose P16INK4a IHC positivity was detected only by Digital Image Analysis through color deconvolution (Figure 3). Out of the 25 remaining “negative” cases, ten were confirmed to be negative also at the digital evaluation, eight showed a barely quantifiable percentage of positive tumor cells, and seven were considered misclassified since they had from 4 to 9% of P16INK4a tumor positive cells. The greater sensitivity and throughput of the Digital Image Analysis approach allowed us to classify a greater number of cases as P16INK4a positive than those assessed by manual evaluation.

**TABLE 4:** Case processing crossing the digital variable by the visual categories.

	Visual Categories	Cases					
		Valid		Missing		Total	
		N	Percent	N	Percent	N	Percent
**Case Processing Summary**							
DIA p16% tum	DP	25	96.2%	1	3.8%	26	100.0%
	Intermediate	29	90.6%	3	9.4%	32	100.0%
	Low	39	97.5%	1	2.5%	40	100.0%
	Negative	27	93.1%	2	6.9%	29	100.0%

**TABLE 5:** Statistical analysis of DIA values across visual categories.

Visual Categories	N	Mean	Median	Std. Deviation	Minimum	Maximum	Std. Error of Mean
**Report**							
DP	25	52.432256	49.236100	18.7965590	26.3976	89.8032	3.7593118
Intermediate	28	28.513707	29.749600	12.1654408	7.6920	55.3540	2.2990522
Low	38	10.761482	7.581100	10.0057792	0.5044	42.9707	1.6231517
Negative	25	2.840236	1.683500	2.9058910	0.0026	9.1280	0.5811782
Total	116	22.320107	17.662400	21.8071546	0.0026	89.8032	2.0247435

*The analysis does not include the four outliers.*

### Correlation of p16 Expression With Clinicopathological Features

**FIGURE 4:**
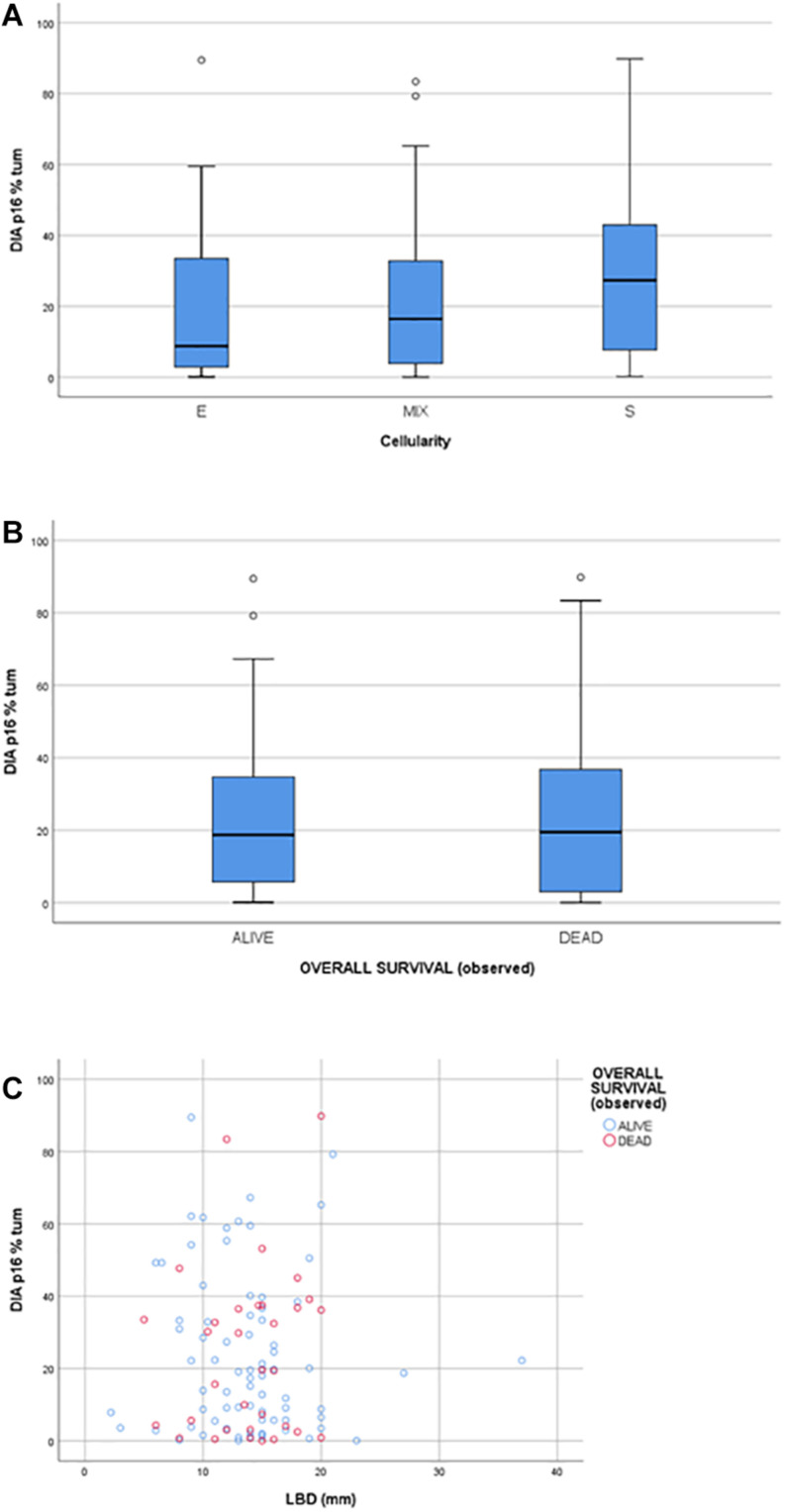
**(A)** box plot showing the distribution of P16INK4a positivity across the Cellularity categories (E: epithelioid, S: spindle; MIX: mixed). **(B)** box plot showing the distribution of P16INK4a positivity between the two Overall Survival categories: alive and dead. **(C)** scatter plot matching P16INK4a positivity and LBD (larger basal diameter).

The statistical analysis ruled out any statistically significant correlation between protein expression and clinical data, including follow-ups (Figure 4B). Moreover, no statistically significant correlation was found between P16INK4a expression and morphological prognostic factors such as the tumor cell cytotype (epithelioid, spindle, or mixed), being the ANOVA statistical test performed between groups not significant (*p* > 0.05); no correlation was found between P16INK4a expression and the largest basal diameter (LBD) (*p* > 0.05) (Figures 4A,C). The area under the ROC curve of DIA derived expression values, related to the outcome, was equal to 0.477 (95% CI: 0.354–0.601) demonstrating a poor prognostic value of DIA p16 percentage (Supplementary Figures 2A,C). The same conclusion was reached performing a Cox regression multivariate analysis, testing DIA p16 values, age, gender, LBD, and cellularity as covariates (Supplementary Figures 2B,D).

## Discussion

Uveal melanoma is a rare neoplasia characterized by unpredictable behavior, and, despite the illusory success of local therapies, more than 50% of patients develop metastases within 5 years from diagnosis (31). Less than 4% of UM patients present metastases concomitant with the primary disease, but the high metastatic rate suggests that clinically undetectable metastases might be present upon diagnosis. Therefore, UM is currently considered a systemic disease (6).

Uveal melanoma has morphological features very similar to CM, especially for cytology. UM cells, like CM cells, may be spindle or epithelioid, from slight to extremely pleomorphic, and present characteristic huge eosinophilic nucleoli. UM cells show immunohistochemical positivity to all the most significant markers of melanocytic differentiation, such as S100, MelanA, HMB45(32), and SOX10 (33). Although UM seems to share with CM the same melanocytic origin, it represents an entirely different neoplasia, especially for the different mutational profiles and the low mutational burden (3). The most frequent mutations of CM, such as BRAF, N-Ras, K-Ras, H-Ras, and NF1, alterations of TERT-promoter or tumor suppressor genes such as ARID2, TP53, PTEN and also CDKN2A (8), have been rarely found in UM and most therapeutic strategies used in CM have proved ineffective in metastatic UM (3). There are no effective systemic treatments for metastatic UM, and the median overall survival, from the diagnosis of metastatic disease, is 12 months (34).

We tested the immunohistochemical expression of the protein P16INK4a in 127 cases of UM and 3 cases of UM metastasis, with both visual and digital approaches. In about 80% of UM cases and all the metastases, the relative amount of antibody-accessible P16INK4a epitope was easily detectable with various grades of expression.

The comparison between visual evaluation and digital assessment of P16INK4a tissue expression revealed a high concordance rate, depending on the visual category. In particular, a digital approach leads to a better definition of the quality of the analyzed material and gives reproducibility and accuracy to the immunohistochemical evaluation (35). Moreover, digital assessment better discriminates immunohistochemical positivity in cases of hyperpigmentation thanks to Digital Image Analysis through color deconvolution. Interestingly, the use of QuPath allowed us to reinterpret the data obtained with the visual evaluation; 24% of the cases visually identified as “negative” showed a digital positivity to P16INK4a of between 3 and 9% of cancer cells. The use of an image analysis software allows sensitivity, precision, and reproducibility that is difficult to obtain through visual analysis.

The finding of P16INK4a expression in a large proportion of UM, confirms the difference between UM and CM, once again. P16INK4a is frequently unexpressed in CM and immunohistochemical analysis of P16INK4a, in combination with Ki67 and HMB45, is useful in the differential diagnosis between nevi and melanoma. Immunohistochemical loss of P16INK4a expression is typical of CM, and the CDKN2A gene is frequently involved in its development (13). Loss of function mutations of the CDKN2A gene are common in familial CM and are reported in 15% of sporadic CM that, instead, present epigenetic downregulation of this gene in 70% of cases (36). It has been hypothesized that CDKN2A gene alterations are early events in CM development and are necessary for the acquisition of invasive potential (8). Neither mutations nor epigenetic alterations of the CDKN2A gene have been described in UM, and this could be the reason for the preserved immunohistochemical expression of P16INK4a in our series. Our cohort reflects high-risk primary UM, as we can see from LBD values, so P16INK4a loss is not a common feature even in large, high-risk, Uveal Melanomas.

P16INK4a expression is present in many tumors from different anatomic sites. Head and neck squamous cell carcinoma, small-cell lung cancer, basal-like breast carcinoma, high-grade ovarian carcinoma, serous uterine carcinoma, and cervical squamous cell carcinoma are all positive for P16INK4a. If in some districts, such as head, neck and cervix, this positivity is related to HPV infection, in other tumors it is probably associated with alterations of the RB pathway (37).

Particularly, a loss of RB induces oncogenic stress with P16INK4a induction. Thus, in the absence of the RB protein, P16INK4a is unable to arrest the cell cycle and consequently tumor progression. RB protein inactivation has been found in UM, due to the phosphorylation of its COOH-terminal region (serine-807/811 and threonine 821), which might also explain expression.

Although mutations of CDKN2A have been rarely described in UM, inactivation of CDKN2A, through promoter methylation or the loss of the 9p region, has been found in one-third of UM and may be involved in UM evolution (23). Recent findings demonstrated that the deletion of CDKN2a could play an important role in the development and metastatic progression in UM, especially with 8q amplification. However, more studies are needed to clarify better the role of this gene in UM development and biological behavior (24, 25).

P16INK4a immunohistochemical expression has never been tested before in such a large population of UM and metastases from UM. Moreover, the finding of P16INK4a expression in UM metastases may be useful to discriminate between UM and CM in cases of metastasis from primary occult malignancy or in patients with multiple primary melanomas. The digital assessment approach of the immunohistochemical expression of the protein undoubtedly represents an important advance in the way of interpreting the tissue expression data, which favors the standardization and reproducibility of the technique.

The molecular basis of P16INK4a expression in UM and its metastases should be better investigated and might provide a series of new therapeutic targets in high-risk and metastatic UM, a clinical context with limited management options.

## Data Availability Statement

The raw data supporting the conclusions of this article will be made available by the authors, without undue reservation.

## Ethics Statement

The study was performed according to the Italian law, and according to the Declaration of Helsinki for studies based only on retrospective analyses on routine archival FFPE-tissue.

## Author Contributions

All authors listed have made a substantial, direct and intellectual contribution to the work, and approved it for publication.

## Conflict of Interest

The authors declare that the research was conducted in the absence of any commercial or financial relationships that could be construed as a potential conflict of interest.

## Supplementary Material

Supplementary Figure 1 The diagram summarizes the workflow of the study. The analyzed samples were taken from the archives of the Pathology Section of the University of Naples “Federico II” and of the University of Catania. TMAs were cored all together. Following P16INK4a immunostaining, the IHC signal was evaluated both visually, and by Digital Image Analysis (DIA) approach, a comparison between the two methods was performed. Both the approaches included a quality check step that excluded some cores lost during processing or not assessable.

Supplementary Figure 2 **(A)** ROC curve of DIA percentage related to outcome (OS = 1, i.e., died of the disease). The table in **(C)** shows the value of the area under the curve and the 95% CI; **(B)** Cox regression model applied to a multivariate analysis, HR are shown in table **(D)**. DIA p16 values were not associated with increased risk of death compared to other clinicopathological covariates such as age, gender, LBD and cellularity. Overall, the significance of the model was weak (*p* > 0.05).
