# Supplementary figures and images for: Expression of P16INK4a in Uveal Melanoma: New Perspectives
Source: Front Oncol. 2020 Oct 13;10:562074. doi: 10.3389/fonc.2020.562074 (PMC7590828; doi:10.3389/fonc.2020.562074)

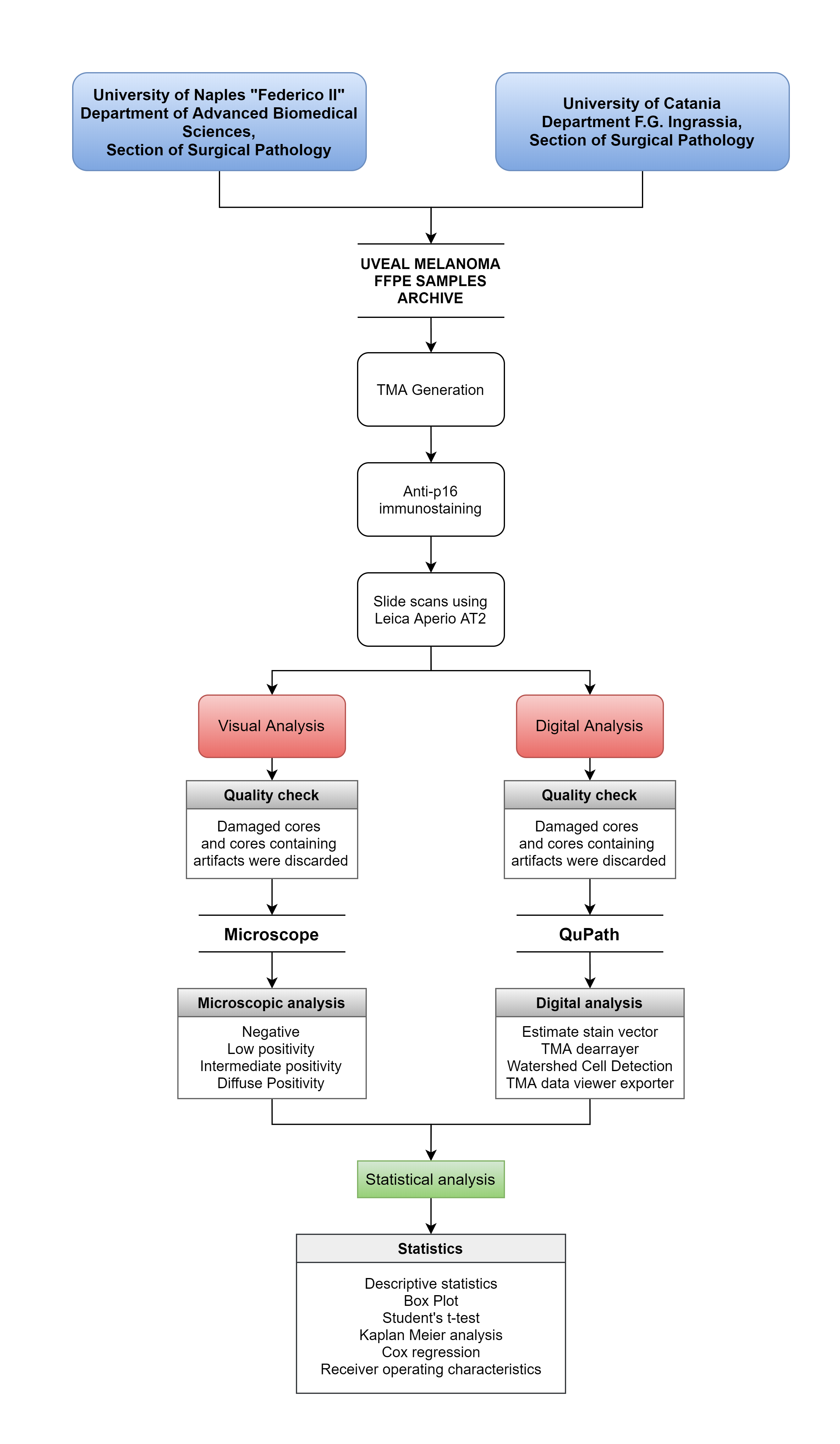

Supplement: Supplementary Figure 1 — The diagram summarizes the workflow of the study. The analyzed samples were taken from the archives of the Pathology Section of the University of Naples “Federico II” and of the University of Catania. TMAs were cored all together. Following P16INK4a immunostaining, the IHC signal was evaluated both visually, and by Digital Image Analysis (DIA) approach, a comparison between the two methods was performed. Both the approaches included a quality check step that excluded some cores lost during processing or not assessable. [file Image_1.TIF]

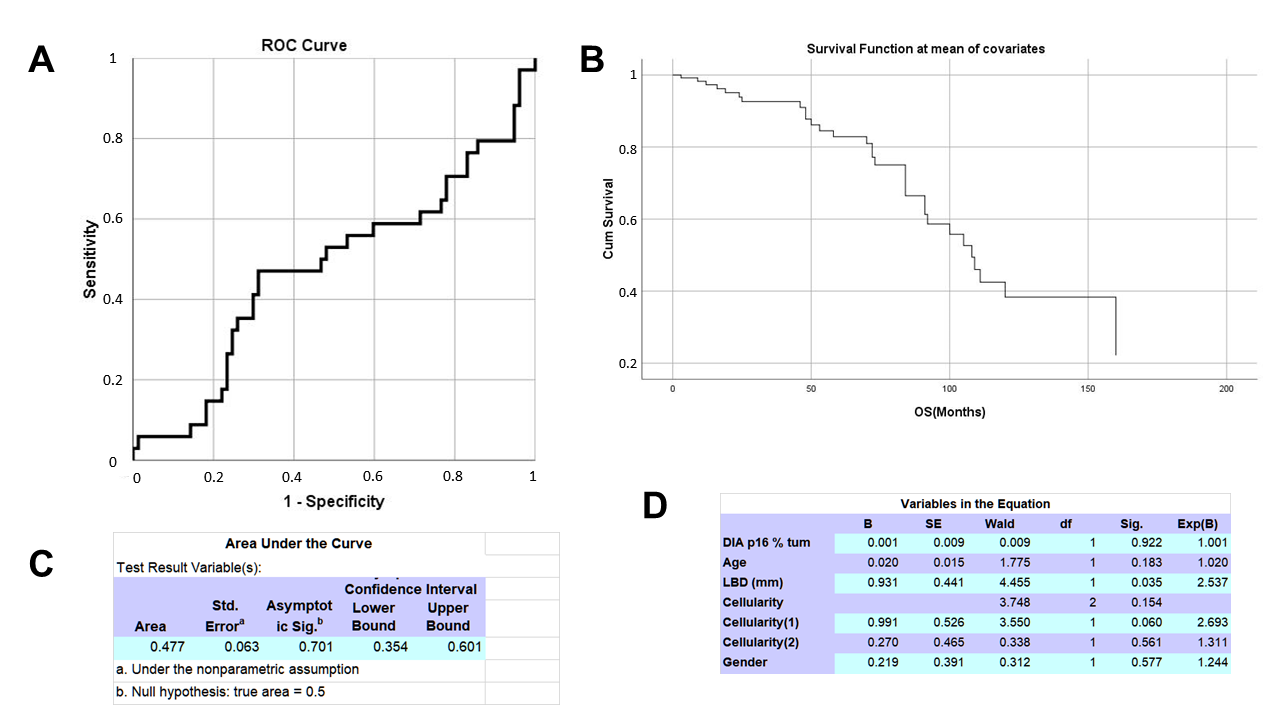

Supplement: Supplementary Figure 2 — (A) ROC curve of DIA percentage related to outcome (OS = 1, i.e., died of the disease). The table in (C) shows the value of the area under the curve and the 95% CI; (B) Cox regression model applied to a multivariate analysis, HR are shown in table (D). DIA p16 values were not associated with increased risk of death compared to other clinicopathological covariates such as age, gender, LBD and cellularity. Overall, the significance of the model was weak (p > 0.05). [file Image_2.TIF]
